# Colloidal nanomaterials for water quality improvement and monitoring

**DOI:** 10.3389/fchem.2022.1011186

**Published:** 2022-09-27

**Authors:** Ana C. Estrada, Ana L. Daniel-da-Silva, Cátia Leal, Cátia Monteiro, Cláudia B. Lopes, Helena I. S. Nogueira, Isabel Lopes, Maria J. Martins, Natércia C. T. Martins, Nuno P. F. Gonçalves, Sara Fateixa, Tito Trindade

**Affiliations:** ^1^ Department of Chemistry and CICECO-Aveiro Institute of Materials, University of Aveiro, Aveiro, Portugal; ^2^ Department of Biology and CESAM-Centre of Environmental and Marine Studies, University of Aveiro, Aveiro, Portugal

**Keywords:** colloids, nanomaterials, surface chemistry, inorganic nanoparticles, water quality

## Abstract

Water is the most important resource for all kind forms of live. It is a vital resource distributed unequally across different regions of the globe, with populations already living with water scarcity, a situation that is spreading due to the impact of climate change. The reversal of this tendency and the mitigation of its disastrous consequences is a global challenge posed to Humanity, with the scientific community assuming a major obligation for providing solutions based on scientific knowledge. This article reviews literature concerning the development of nanomaterials for water purification technologies, including collaborative scientific research carried out in our laboratory (nanoLAB@UA) framed by the general activities carried out at the CICECO-Aveiro Institute of Materials. Our research carried out in this specific context has been mainly focused on the synthesis and surface chemical modification of nanomaterials, typically of a colloidal nature, as well as on the evaluation of the relevant properties that arise from the envisaged applications of the materials. As such, the research reviewed here has been guided along three thematic lines: 1) magnetic nanosorbents for water treatment technologies, namely by using biocomposites and graphite-like nanoplatelets; 2) nanocomposites for photocatalysis (e.g., TiO_2_/Fe_3_O_4_ and POM supported graphene oxide photocatalysts; photoactive membranes) and 3) nanostructured substrates for contaminant detection using surface enhanced Raman scattering (SERS), namely polymers loaded with Ag/Au colloids and magneto-plasmonic nanostructures. This research is motivated by the firm believe that these nanomaterials have potential for contributing to the solution of environmental problems and, conversely, will not be part of the problem. Therefore, assessment of the impact of nanoengineered materials on eco-systems is important and research in this area has also been developed by collaborative projects involving experts in nanotoxicity. The above topics are reviewed here by presenting a brief conceptual framework together with illustrative case studies, in some cases with original research results, mainly focusing on the chemistry of the nanomaterials investigated for target applications. Finally, near-future developments in this research area are put in perspective, forecasting realistic solutions for the application of colloidal nanoparticles in water cleaning technologies.

## 1 Introduction

Research on colloidal inorganic nanomaterials has developed considerably in the last decades. Taking the last century transition period as a yearly timeline reference, it is possible to trace to about that period the development of important chemical methods for the synthesis of quantum dots, magnetic metal oxides and metals, just to mention a few relevant classes of nanomaterials (Brus, 1984; Ekimov et al., 1985; Bawendi et al., 1990; Steigerwald and Brus, 1990; Schmid, 1992; Weller, 1993; Alivisatos, 1996; Leslie-Pelecky and Rieke, 1996; López-Quintela and Rivas, 1996; Trindade and O’Brien, 1996; Weller, 1998; Manna et al., 2000; Peng and Peng, 2001; Trindade et al., 2001; Esteves and Trindade, 2002; Frey et al., 2009; Teitelboim et al., 2016). Associated to the development of synthetic methods of nanosized colloids has emerged a new paradigm in inorganic synthesis concerning the control of size and shape-dependent intrinsic properties of colloidal particles (Jun et al., 2006; Grzelczak et al., 2008; Tao et al., 2008; Xia et al., 2009; Guerrero-Martínez et al., 2011; Lim and Xia, 2011; Watt et al., 2013; Zhang et al., 2013). Indeed, it is at the nanoscale that several crystalline solids show the transition of the bulk-type behaviour to a cluster-like behaviour as particle size decreases, which scales with a critical dimension identified for that type of solid. Hence, it is now routine to employ chemical methods for preparing semiconductor nanocrystals, whose band gap can be tuned by adjusting the particle size distribution below a certain threshold defined as the Bohr radius of that semiconductor or to synthesise superparamagnetic iron oxides whose dimensions are below the size of Weiss-domain. The control of the optical, electronic and magnetic properties of materials using size (and shape) as additional parameters of chemically controlled characteristics of particles has important consequences in advancing the frontiers of knowledge and on new applications of materials, which is one of the reasons, among others, for which Chemistry is regarded as a central science also in Nanotechnology (Whitesides, 2005; Yina and Talapin, 2013). Furthermore, the properties, manipulation, processing and applications of colloidal inorganic nanoparticles are markedly dependent on their surface chemistry, which has been an active research topic in the last 2 decades, including in our laboratory (Yin and Alivisatos, 2005; Boles et al., 2016; Daniel-da-Silva and Trindade, 2021).

The unique properties of inorganic nanoparticles make them suitable for applications in diverse areas, which include nanomedicine (e.g., drug delivery, bioimaging, phototerapies) (Lin et al., 2007; Sokolova and Epple, 2008; Trindade and Daniel da Silva, 2011; Vega-Vásquez et al., 2020; Wang and Mattoussi, 2020; Ray and Bandyopadhyay, 2021), energy (e.g., solar cells, lightning) (Kamat, 2008; Cheng et al., 2016; Kumar et al., 2017; Chen et al., 2018; Choe et al., 2021), environment (e.g., water and air purification (Theron et al., 2008; Kumari et al., 2019; Weon et al., 2019; Wadhawan et al., 2020; Li et al., 2021), greenhouse gases capture (Kumar et al., 2020; Cheng et al., 2021), sustainable technologies (e.g., catalysis, biological synthesis) (Polshettiwar and Varma, 2010; Dutta et al., 2015; Kumari et al., 2020; Jeevanandam et al., 2022; Liu et al., 2022; Wani and Suresh, 2022), among many others. These endeavours require transdisciplinary approaches, among which the synthesis and surface modification of the materials are usually regarded at the upstream in the supply chain of functional materials for such devices and processes. A main aspect of our late research has been the exploration of chemical methods for modifying the surface of inorganic nanoparticles, as shown below with distinct systems. Mostly of these methods rely on colloid chemistry of inorganic particles establishing important bridges to other scientific domains, such as polymer chemistry and coordination chemistry. In this context, a series of functional inorganic nanoparticles have been investigated, depending on the target application.

Several water cleaning technologies have been developed, including adsorption, photocatalysis and membrane filtration. Adsorption is an efficient method, very attractive due to the affordable cost and easy operation. Photocatalysis enables water treatment in an eco-friendly manner because solar radiation can be used as an energy source. However, complete mineralization of the pollutants is challenging and may result in oxidation by products formation. The interest in membrane filtration technologies has grown significantly in the last years, but it still faces challenges in membrane fouling, concentrate disposal and high energy consumption. Together with remediation technologies, preventing measures should be also implemented namely by developing water quality monitoring protocols, that can be easily accessed in different regions of the globe. As an example of this approach, several sensors have been investigated, namely envisaging the optical detection of water contaminants using low-cost and disposable substrates. In this work, we focus on the research of colloidal inorganic nanomaterials and their composites, aiming at nanotechnologies for water cleaning and quality monitoring, which has been a common ground for some of the research carried out in our laboratory during the past years.

## 2 Nanomaterials for water cleaning nanotechnologies

### 2.1 General framework

Water and wastewater management is a major global concern considering an ever-increasing population, Human-driven water pollution and the scarcity of freshwater resources. Specifically, water pollution is critical since it may cause negative impacts on ecosystems and Human health, and it can as well adversely impact economic growth and the social perspectives of societies. Considering this framework, the Organization of United Nations (UN) acknowledged that the availability of good water quality is vital for all living creatures and must be considered a worldwide challenge for this century. The preface of the last edition of the “Guidelines for drinking-water quality” published by the World Health Organization begins with the undisputable sentence: “Access to safe drinking-water is essential to health, a basic human right and a component of effective policy for health protection” (WHO, 2022). Among the seventeen Sustainable Development Goals agreed by world leaders, the Goal 6 (SDG 6) aims to ensure access and sustainable use of water for everyone, while SDG 15 aims to protect, restore and promote sustainable use of ecosystems. Water contamination is a global problem affecting populations and ecosystems, putting at risk such goals, namely by limiting the access to safe drinking-water of large populations worldwide. The prioritisation of this issue has increased in the past decades due to several factors, which includes accentuated water scarcity due to climate changes and increasing levels of water contaminants of emerging concern (CEC). The complexity of this global problem requires actions and decisions at very diverse levels, engaging different actors, from the ordinary citizen to policy decision makers and, with obvious social responsibility, the scientific community.

In this context, several nanotechnologies have been developed with potential for implementation and contribute for water remediation and quality improvement. Most of these procedures rely on the use of nanoengineered materials, which have been surface modified accordingly to the envisaged application and the target contaminants to be eliminated. Probably future developments in this area should be better considered as complementary to conventional water cleaning technologies, which for several purposes are quite effective but less so for the elimination of CEC in certain aqueous environments. Also, water quality monitoring technologies that are easily implemented and communicated in different parts of the globe, including in remote regions, are regarded as important advancements, namely because they value prevention behaviour in relation to remediation practices.

**FIGURE 1:**
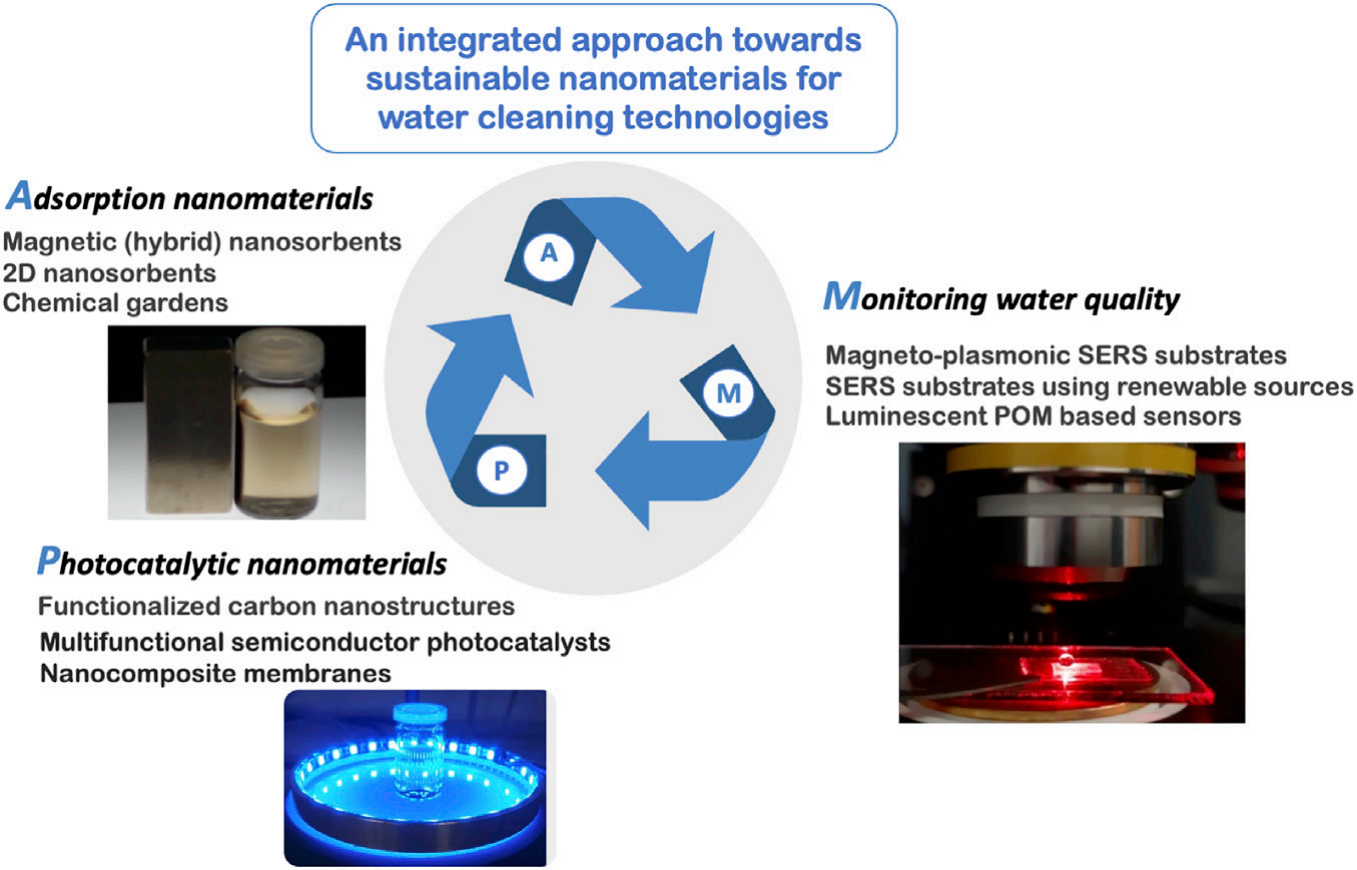
Research topics on sustainable nanomaterials for improving water quality by Adsorption, Photocatalysis and Monitoring by SERS sensors.

The following article sections are built upon research lines that converge in providing colloidal nanomaterials for water quality improvement and monitoring. Figure 1 illustrates these thematic lines, which have been investigated in our research activity during the past years, as well as new research lines. Rather than providing an exhaustive literature survey on each topic, we favour the communication of our own perspectives on nanomaterials developments that have a great of deal to contribute for water cleaning nanotechnologies as identified in illustrative cases of study. Ideally, clean water for all should be supported in sustainable and clean technologies, favouring water contamination prevention rather than remediation measures, but in fact contaminated water is a real problem and purification nanotechnologies can contribute for its solution. The next paragraph provides a summary of topics approaching both general strategies, by following Figure 1 as an illustrative guide.

Adsorption techniques applied to water purification are well-established and carbon activated sorbents are ubiquitous in several procedures. However, the removal of certain water pollutants can be ineffective by using such processes, which requires non-conventional approaches. Surface modified nanosorbents for magnetic separation technologies and multifunctional nanostructures for photocatalysis are examples of nanomaterials that might have impact on new technologies for CEC removal or its prevention. Under this scope, chemical gardens have also attracted our attention. These are self-organizing structures created by nonequilibrium processes and have been known since the discoveries made by Johann Glauber (1646). Still, chemical gardens hide new possibilities for technological applications, including selective adsorption-desorption processes. It is known that these micro- or nano-tubules have internal reactive surfaces whose chemistry can be explored to develop sorbents for CEC.

Contexts of applications are diverse but typically might include water treatment stations of industries, hospitals, and municipalities. These applications are clearly dependent on the functionality and associated efficacy of the nanoengineered materials, for which surface chemistry plays a major role. The presence of CEC in water, even in vestigial amounts, poses serious threats to human health and jeopardizes the access of populations to safe drinking-water. Thus, not only water purification methods effective for pollutants’ diminutive amounts are required, but accessible and sensitive sensors for water quality monitoring would be a step forward for management of water supplies. Nanotechnology provides a myriad of possibilities for fabricating reliable sensors, which ideally should also be low-cost and user friendly. Together with technologies based on the internet of things, these sensors might offer on-time answers for water contamination events which might occur in remote zones. As it happens for water remediation and purification technologies, the nanosensors’ performance depends on the materials used, namely on their selectivity and nanoscale properties that can be explored for achieving lower detection limits in the field analysis. Optical sensors based on low-cost and versatile SERS substrates are among the most promising for several applications in this context. The risk-benefit balance of these nanomaterials in water cleaning technologies is crucial to decide on their practical use in real contexts. Studies on the assessment of the impact on health and environment of these materials have been carried out, providing a wealth of data that requires a critical and thoroughly analysis. Our research in this area has been mostly collaborative, in providing nanomaterials that have been found as promising for experts on nano- and eco-toxicology to assess, as is also illustrated in this work as an important part of research in this area.

### 2.1 Functionalized magnetic nanosorbents

#### 2.2.1 Biocomposite particles

Biopolymers, i.e., natural polymers produced by living organisms, are sustainable materials with low toxicity, abundant and available, whose structure contains functional groups with an affinity towards diverse pollutants. The properties of biopolymers and their composites have prompted a focus on water treatment applications, namely as adsorbents for water decontamination (Nasrollahzadeh et al., 2021; Yaashikaa et al., 2022). One of the major issues with the adsorption-treatment process is the recovery of spent sorbents, which should be easy and cost-effective (Baskar et al., 2022). Combining biopolymers with magnetic iron oxide nanocrystals results in composites that are easily recovered using magnetic separation, making them very appealing for water treatment through adsorption (Abdel Maksoud et al., 2020; Soares et al., 2020). A rational modification of the surface of the magnetic nanocrystals with the biopolymer is necessary to attain pollutant specificity, high adsorption capacity and reusability. For example, covalent immobilization of the biopolymer onto the surface of the particles is critical to ensure the successful recycling and reuse of the biosorbents.

**FIGURE 2:**
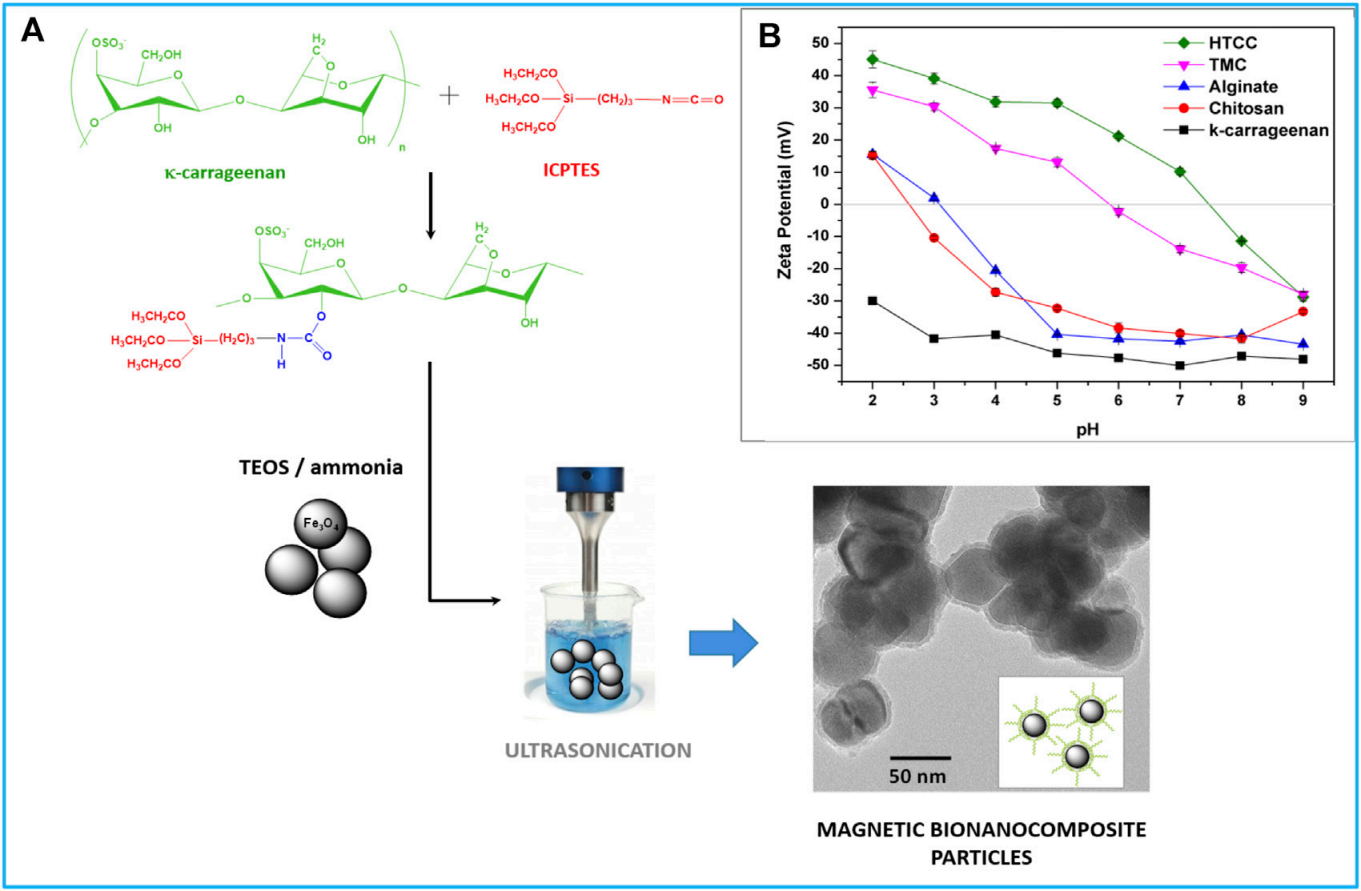
**(A)** Scheme illustrating the chemical route to prepare the silicon alkoxide derivative of the biopolymer k-carrageenan and the preparation of the magnetic biosorbents through coating the Fe_3_O_4_ nanoparticles with the bio-hybrid siliceous shell; **(B)** Potential zeta values of magnetic biosorbents prepared using distinct biopolymers (HTCC -N-(2-hydroxypropyl)-3-trimethylammonium chitosan; TMC- trimethylchitosan).

Our group has developed a method for preparing magnetic biosorbents that took in consideration the context above. The biopolymer is grafted onto a siliceous network that acts as a surface coating of Fe_3_O_4_ nanoparticles during the encapsulation process, yielding an organic-inorganic hybrid shell enriched in the biopolymer component. The encapsulation encompasses the hydrolysis and condensation of a mixture of tetraethyl orthosilicate (TEOS), as the SiO_2_ precursor, and a silicon alkoxide derivative of the biopolymer, in the presence of colloidal magnetic particles. Figure 2A illustrates the preparation of the magnetic biosorbents using the polysaccharide κ-carrageenan, encompassing the derivatization of the biopolymer with a silane coupling agent functionalized with isocyanate groups (3-isocyanatopropyl triethoxysilane-ICPTES) and the subsequent coating of the Fe_3_O_4_ nanoparticles. This method originates more robust coatings and with a higher degree of functionalization when compared to the conventional techniques of non-covalent coating and surface-grafting approach, which is desirable to obtain reusable sorbents with improved adsorption capabilities. A series of magnetic biosorbents have been prepared by using this route, from polysaccharides with distinct ionic character and variable chemical functionalities, which were efficient in the uptake of target pharmaceuticals (Soares et al., 2016; Soares et al., 2019a; Soares et al., 2019b; Soares et al., 2019c; Soares et al., 2021b; Soares et al., 2022), pesticides (Fernandes et al., 2017; Soares et al., 2021a), organic dyes (Soares et al., 2017b), and non-polar organic solvents from water (Soares et al., 2017a). The biopolymer selected allows the preparation of magnetic nanosorbents with various chemical functionalities and tunable surface charge (Figure 2B). This is a crucial step to attain high adsorption capacity because electrostatic interactions between oppositely charged groups of the biopolymer and the pollutant species play an essential role in their adsorption. Nevertheless, other interactions, such as H-bonding and hydrophobic interactions, may also contribute to the adsorption process (Crini et al., 2019; Badsha et al., 2021). Assessing the adsorptive performance in conditions closest to the final application is of utmost importance in developing effective treatments. This assessment frequently involves testing the removal of the pollutant in trace concentrations in aqueous matrices of complex nature. In this context, magnetic biosorbents prepared from trimethyl chitosan-based decreased environmentally realistic concentrations of the pesticide glyphosate in spiked effluents of wastewater treatment plants up to 80%, demonstrating their potential application in real conditions (Soares et al., 2021a).

#### 2.2.2 Carbon-based 2D materials

Carbon is one of the most abundant elements in the environment and human body (Rao et al., 2021), with the particularity of having an extraordinary ability to bind to other elements. Carbon has extensive allotropy but, until the eighties, few carbon allotropic forms were known, namely, diamond, graphite and fullerene (Kallumottakkal et al., 2021). Since then, many other allotropes have been discovered and produced, including ball shapes such as buckminsterfullerene and sheets such as graphene, opening a research window for a variety of carbon nanomaterials of distinct structural dimensionality.

Two-dimensional nanomaterials are considered the thinnest nanostructures among the different dimensional groups, and contrary to bulk materials, have a high surface-area-to-volume ratio (Kallumottakkal et al., 2021). In the 2D nanostructures, the relative number of external to internal atoms is higher, and their different functions lead to a change in the behavior of 2D nanomaterials, in comparison to the other dimensional groups, conferring them a variety of unique physical, chemical, and electrical properties (Rafiei-Sarmazdeh et al., 2019). Graphene is a 2D material with a hexagonal structure composed of sp^2^-hybridized C atoms, where each carbon atom is covalently bound with each other in the same plane (Kallumottakkal et al., 2021). For water compatible applications, pristine graphene faces some problems such as poor solubility and agglomeration tendency (Joseph et al., 2021). To overcome these limitations, researchers have come up with a handful of techniques to synthesize structurally similar compounds, like graphene oxide (GO) and graphite-like nanoplatelets (GNP), which can be produced from carbon sources by simple top-down methods (Rao et al., 2021).

Our research group has been interested in exploring GNP for their adsorption capability and ability to enhance the adsorption capacity of various composites, with the aim of removing toxic metal ions from water and/or recovering valuable elements, such as technology critical elements. There has been great interest in this topic (Cardoso et al., 2019) because emerging key technologies are intrinsically dependent of a limited number of elements often termed by technology critical elements (TCE), which includes all lanthanoids and the platinum group elements (Pinto et al., 2021). GNP are a viable and inexpensive materials that can be used as stable substrates for preparing magnetic nanocomposites for water adsorption treatment, thus allowing their use in magnetic separation technologies, as already discussed in the previous section. The GNP are decorated with nanoparticles of spinel ferrites (MFe_2_O_4_, M = Fe, Mn, Co.), thereby conferring ability for magnetic separation of the sorbents when exposed to an external magnetic gradient.

**FIGURE 3:**
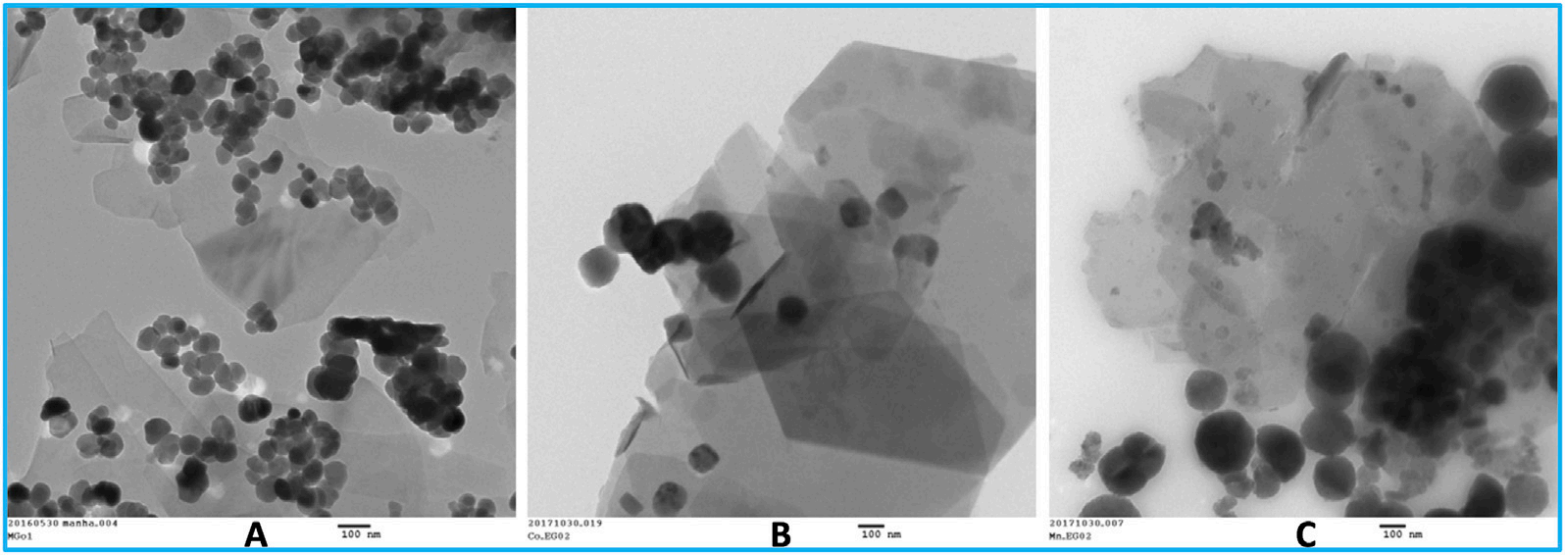
TEM images of nanocomposites: **(A)** Fe_3_O_4_/GNPs; **(B)** CoFe_2_O_4_/GNPs and **(C)** MnFe_2_O_4_/GNPs.

The magnetic nanostructures have been prepared by *in situ* alkaline hydrolysis or electrostatic assembly of particulates previously synthesized. Figure 3 shows TEM images of spinel ferrites, with average sizes below 100 nm, attached onto GNP substrates. All materials quickly approach saturation in the presence of an external magnetic field, and the estimated saturation magnetization, at 300 K, is about 45 emu/g for Fe_3_O_4_/GNP and 29 emu/g for the CoFe_2_O_4_/GNP and MnFe_2_O_4_/GNP composites. As expected, these estimated saturation magnetization values are lower than the values of the respective spinel ferrites at room temperature (Fe_3_O_4_ 92 emu/g, CoFe_2_O_4_ 55 emu/g and MnFe_2_O_4_ 48 emu/g).

The Fe_3_O_4_/GNP nanocomposites were investigated to capture selected lanthanoids (La, Eu, and Tb), from unary and ternary solutions, and their performance was compared with the one observed for the isolated components (Fe_3_O_4_ and GNPs) (Afonso et al., 2019). After a 24 h period of exposition of equal amounts of GNP, Fe_3_O_4_ NPs and Fe_3_O_4_/GNPs to a ternary solution of La(III), Eu(III) and Tb(III), with an elemental concentration of 100 μg/L, the results show distinct affinity between materials and elements, with the Fe_3_O_4_/GNPs composite showing better capability to remove the target elements from water than any of its counterparts (La: 4.7% (GNPs), 3.1% (Fe_3_O_4_ NPs), and 15% (Fe_3_O_4_/GNPs); Eu: 1.2% (GNPs), 0.2% (Fe_3_O_4_ NPs) and 37.6% (Fe_3_O_4_/GNPs); Tb: 0.2% (GNPs), 0.1% (Fe_3_O_4_ NPs) and 35.0% (Fe_3_O_4_/GNPs)). The better performance of the nanocomposites can be explained by an increase of active sorption sites due to the introduction of oxygen moieties into the carbon lattice. Trivalent lanthanides are hard Lewis acids with strong chemical affinity for oxygen donors. More, the sorption process showed to be very dependent from solution pH, and only feasible for pH values higher than 5.2, which is the experimental isoelectric point of the composite (Afonso et al., 2019). Consecutive sorption and desorption cycles were also applied to ternary solutions of TCE (100 μg/L) and for the best experimental conditions studied (pH 7.5, 250 mg/L of Fe_3_O_4_/GNPs) and using 0.1 M HNO_3_ as eluent solution. During the consecutive cycles, the recovery efficiency of the target elements ranged from 82 to 92% for La (III), 88–104% for Eu (III) and 82–97% for Tb (III). These data elucidate that dilute nitric acid solution (0.1 mol/L HNO_3_) is an efficient eluent for the recovery of these elements from the magnetic composite. More, the capability of this nanostructure to remove the selected TCE from water was rarely affected since the values of the removal efficiency remained nearly constant during all cycles, also confirming the stability of the magnetic composite.

In other study, Fe_3_O_4_/GNPs, CoFe_2_O_4_/GNPs and MnFe_2_O_4_/GNPs composites were investigated to capture mercury and arsenic, from unary and binary solutions. Among the toxic substances of anthropogenic and natural origin, these elements pose greatest threat to the environment, due to their non-degradable nature, severe toxicity and bioaccumulative character (Lopes et al., 2008; Tavares et al., 2020). Arsenic and Hg occupy the 1st and 3rd positions, respectively, on the list of priority hazardous substances of 2019 provided by the Agency for Substances and Toxic Diseases, and so their effective removal from water is a priority to achieve the SDG6 of United Nations. The pH dependency, of the sorption process using these magnetic nanostructures was assessed for the range 4–9. After a 24 h period of exposition of equal amounts of MFe_2_O_4_/GNPs, to unary solutions of As(III) (1000 μg/L) and Hg(II) (50 μg/L), the results showed that 1) Fe_3_O_4_/GNPs were the least effective for removing As(III) or Hg(II) and, 2) As(III) removal was higher for the CoFe_2_O_4_/GNPs at pH 7 (90.4%), while MnFe_2_O_4_/GNPs displayed higher efficiency to remove Hg(II) at pH 6 (90.6%). In binary solutions, CoFe_2_O_4_/GNPs showed more efficiency to As(III) with removal percentages near 90% in ultra-pure water and around 80% in tap water, while MnFe_2_O_4_/GNPs removed preferably Hg(II) (85%) in both matrices. Although the above nanocomposites were prepared by similar methods, those containing Co and Mn ferrites performed better than the Fe_3_O_4_/GNPs but on the other hand show more potential for metal leaching (Tavares et al., 2020). Indeed, the ferrites nanoparticles display different metal leaching (20–50% of Co, 20–30% of Mn, and <8% of Fe). The free Mn, Co, or Fe sites located at the surfaces of the respective ferrites could exchange with ions in solution, working as active sorption sites, and the different degree of leaching could also explain the different affinity toward the ions.

These studies highlight that magnetic graphite-like nanoplatelets composites can be explored as effective sorbents for different elements. The nanocomposites are prepared using a simple and environmentally friendly procedure, with potential for future application in the removal of toxic elements and/or in the recovery of valuable elements, such as TCE, from industrial or other water systems.

### 2.3 Nanocomposites for photocatalysis

#### 2.3.1 Metal oxide nanophotocatalysts

TiO_2_ has been the most used semiconductor photocatalyst for effluent treatment because it is relatively inexpensive, photostable over a wide range of pH and the photo-generated hole-electron pairs are efficient in producing highly reactive oxygen species (Kubacka et al., 2012). In particular, TiO_2_ colloidal nanoparticles have several advantages because of their high surface-to-volume ratio, increased number of delocalized charge carriers over the surface, improved charge transport and lifetime afforded by their dimensional anisotropy, and efficient contribution to the separation of photo-generated holes and electrons (Wang et al., 2014). Previous studies have shown that adsorbable organic halides (AOX) produced in the bleaching treatment stage in pulp and paper mills, can be photodegraded under UV radiation by using TiO_2_ or ZnO photocatalysts (Yeber et al., 2000; Ugurlu and Karaoglu, 2009; Kumar et al., 2011). Typically, the metal oxides are dispersed in the effluent as colloids and, at the end of the treatment, the separation and recovery of the photocatalysts are required, which in practice is a costly task and also poses raises issues concerning their effective separation. As discussed below, the immobilization of the catalyst in active supports is a promising strategy, but for conventional immobilized systems there is usually less efficiency in photodegradation due to a reduction in the specific surface area of the catalyst (Li et al., 2009). Alternative strategies have been investigated, such as coupling TiO_2_ semiconducting phases to magnetic nanostructures, enabling magnetic separation of the hybrid photocatalysts without need additional downstream treatment processes. However, these strategies have been mostly tested in degrading dye pollutants in synthetic solutions (Li et al., 2009; Abbas et al., 2014; Gómez-Pastora et al., 2017). There is a lack of studies regarding the applicability of magnetic TiO_2_ hybrids in the treatment of real effluents, namely regarding photodegradation of AOX in samples from pulp and paper mills. The following paragraphs in this sub-section describe our research on this scientific problem by using real effluents provided by an industrial unit localized in the Aveiro (Portugal) region.

First, the TiO_2_ photocatalysts were designed for their magnetic properties through the co-precipitation of magnetite (Fe_3_O_4_) in the presence of P25 TiO_2_. Magnetic water-dispersible TiO_2_ nanostructures, with a magnetite content of 86 wt% (hybrid A) and 67 wt% (hybrid B), have been prepared by varying the reactants ratio. The ensuing hybrid nanostructures were composed of spheroidal magnetite 12 ± 2 nm in diameter and a TiO_2_ nanoparticles (24 ± 9 nm), in a ratio 80/20 wt% of anatase/rutile phases. The Fe_3_O_4_/TiO_2_ hybrids were characterized by an absorption edge in the visible region, which is dominated by the Fe_3_O_4_ adsorption whose direct band-gap energy is 2.27 eV. The magnetic TiO_2_ hybrids were then tested for degrading AOX compounds present in the effluent of the bleaching stage from Kraft pulp mills (pH = 2), under UV light irradiation in the presence of H_2_O_2_ as the co-catalyst.

**FIGURE 4:**
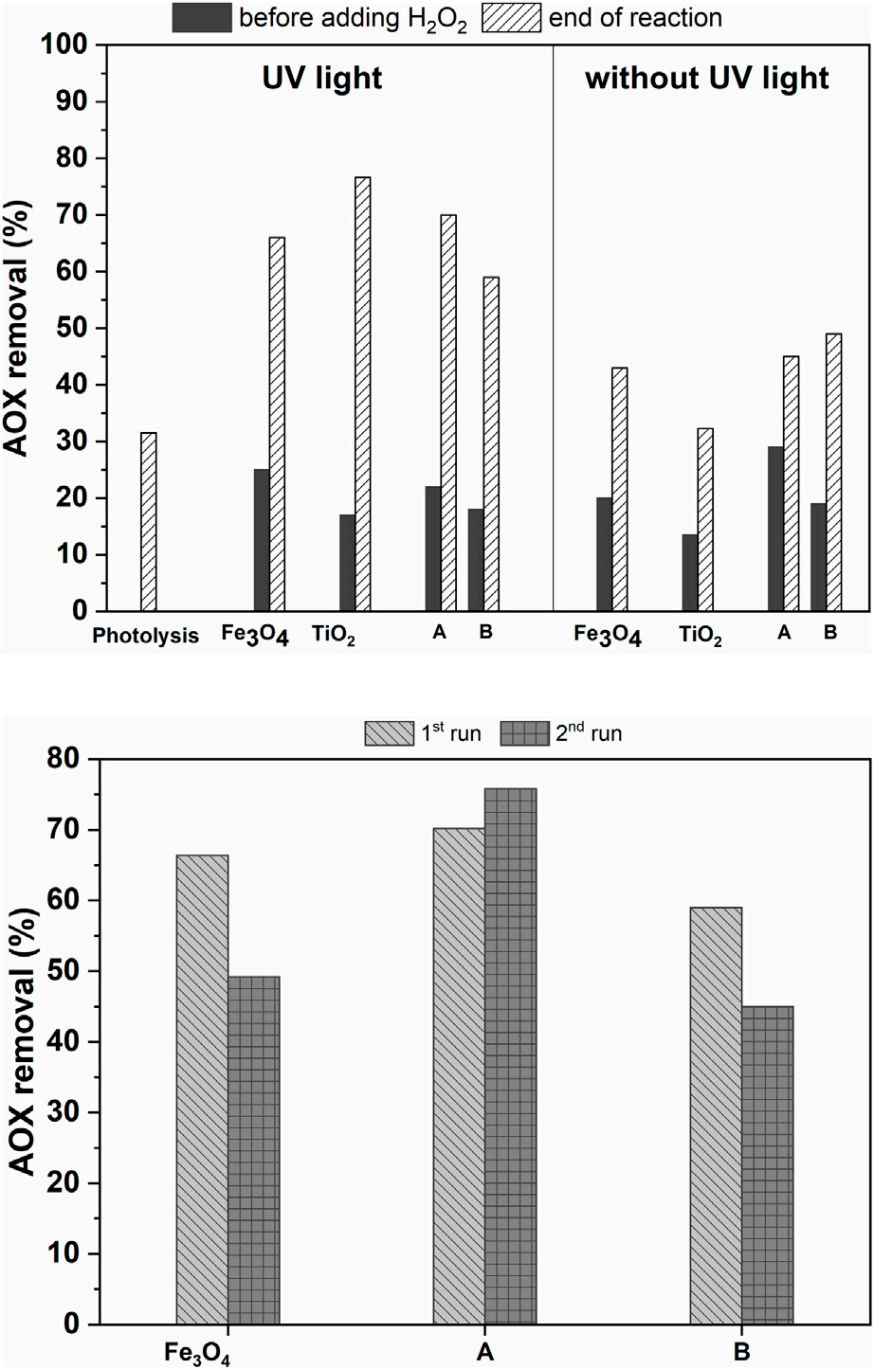
AOX removal in a real aqueous effluent as a function of photocatalyst used (A) in the presence or absence of UV light irradiation (top) and in two consecutive photocatalysis runs using Fe_3_O_4_ and Fe_3_O_4_/TiO_2_-A and B photocatalysts (bottom). Reaction conditions: 2 mg of photocatalyst; AOX effluent (200 ml); 30 wt%, H_2_O_2_ (9.8 mmol); 57°C; 1 h reaction time.

AOX photodegradation was observed regardless the amount of TiO_2_ content in the hybrid photocatalyst. Although the highest AOX removal was observed when using non-magnetic TiO_2_, the magnetic hybrids enabled 59–73% AOX removal after 60 min under UV irradiation (Figure 4), with the great advantage that could be separated from the treated effluent by magnetic separation and subsequently reused. The identity of the hybrids was intact after two photocatalytic cycles, even though a slight loss of photocatalytic activity and mass loss was observed.

#### 2.3.2 POM supported photocatalysts

Polyoxometalates (POMs) show multi-advantages for application as photocatalysts (Walsh et al., 2016), namely their switch-ability by visible light, the possibility of multi-electronic photoreduction, followed by reversible electron exchange with a substrate, the possibility of heterogenization and finally a low cost. The possibility of heterogenization without loss of POM properties is very important for the application of POM based nanomaterials in photocatalysis.

In this work we have been developing new nanostructured hybrid materials based on polyoxometalate clusters supported in graphene (Granadeiro et al., 2012). Graphene supports were obtained by graphene oxide reduction (rGO, reduced graphene oxide). These materials have been optimized for application in the photocatalytic degradation of water pollutants of pharmaceutical origin, one of the most important classes of emergent environmental contaminants (Lopes et al., 2021). The new electron transfer features resulting from the synergetic combination of POMs and rGO have been explored in photocatalysis.

**FIGURE 5:**
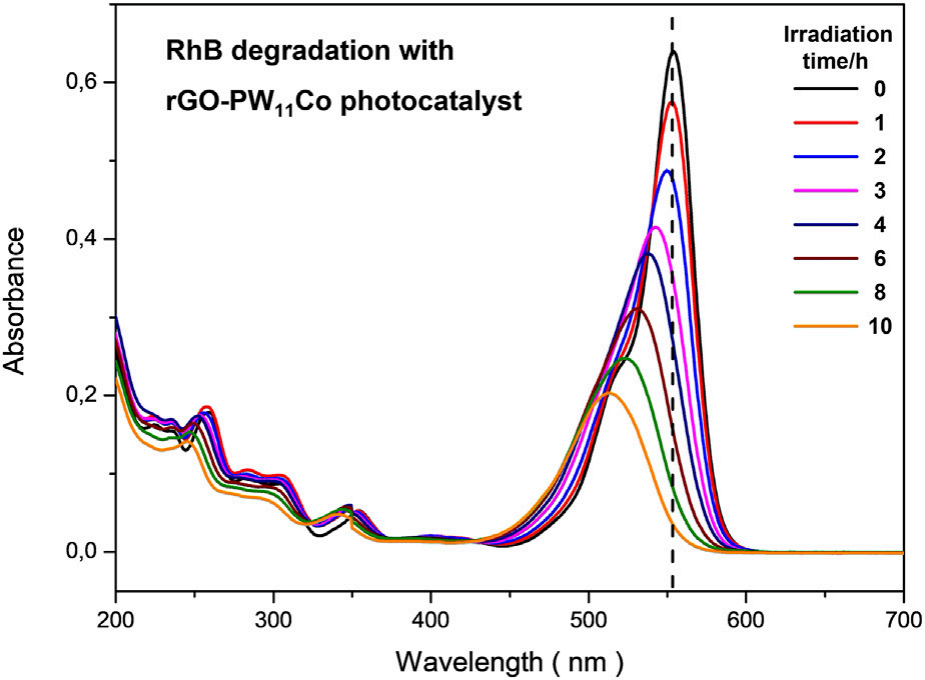
Absorption spectra recorded during the photocatalytic degradation of Rhodamine B (10 ppm) in the presence of 14 mg of rGO-PW_11_Co under irradiation of a 400 W halogen lamp.

To evaluate the photocatalytic activity in the visible of the polyoxometalate [PW_11_CoO_39_]^5−^ supported in rGO (rGO-PW_11_Co), the photodegradation of Rhodamine B (RhB) under visible-light irradiation was conducted at room temperature. RhB was selected as an organic dye model because it shows strong absorption in the visible region (maximum at 554 nm, Figures 5, 0 h) and is photostable in the absence of photocatalyst. As control, identical experiments in the dark were also carried out. The results for a blank experiment, without catalyst, demonstrate that RhB concentration was practically unchanged, suggesting that RhB is stable under visible-light irradiation. Comparative photocatalytic studies using as catalyst the supported rGO-PW_11_Co material and the pure solid K_5_ [PW_11_CoO_39_], respectively, under the same experimental conditions, demonstrate that rGO-PW_11_Co shows higher photocatalytic activity than the polyoxometalate by itself. The photodegradation of RhB is closed to 95% after 600 min in the presence of rGO-PW_11_Co catalyst, under visible-light irradiation. The degradation of RhB is followed by the decrease in the characteristic absorbance at 554 nm as well as the maximum absorption shift over time (Figure 5).

The later example presents a POM based heterogeneous photocatalyst (rGO-PW_11_Co) that shows activity under promising conditions, in particular the use of visible-light for irradiation and room temperature.

#### 2.3.3 Nanoparticles immobilized in/on membranes

Although photoactive nanoparticles have demonstrated effectiveness for the removal of CEC in wastewater, their direct application as free nanoparticles in suspension mode has also limitations. Nanoparticles tend to aggregate with time, due to Van der Walls interactions, which decrease their performance (Lofrano et al., 2016). Most importantly it requires a complex post-treatment step for the recovery/separation of the solid slurry, with a substantial impact on the overall process cost (Loeb et al., 2019). Thus, more effective strategies have been explored based on the immobilization of nanoparticles in/on membranes (Shen et al., 2020). Besides the fundamental economic advantage, coupling nanoparticles with membranes allow easy scaling and reusability with fewer environmental risks, thus contributing to their real application in wastewater treatments. In these systems, the membrane has the simultaneous role of catalyst support while acting as a barrier for the pollutants to be degraded (Iglesias et al., 2016). Retained molecules during filtration, depending on their physical and chemical properties, are forced towards the membrane increasing their concentration near the liquid/catalyst interface, thus boosting the degradation rate (Presumido et al., 2021). Moreover, nanocomposite membranes are of interest as it also reduces the membrane fouling effect, which remains the notorious drawback of micro- (MF), ultra- (UF) and nanofiltration (NF) (Xu et al., 2020).

The introduction of nanoparticles promotes changes in morphology and enhances membrane hydrophilicity due to their polarity. Following the solution–diffusion theory, an increase in the nature of the membrane benefits water diffusion and controls the transport process through the membrane, improving its permeability. Conversely, immobilized nanoparticles have reduced active surface area, which entails the maximization of crucial parameters such as light irradiation and mass transfer (Alipour Atmianlu et al., 2021). Different oxides nanoparticles (photocatalysts) have been explored, such as TiO_2_ (Martins et al., 2016), ZnO (Shen et al., 2020), ZrO_2_ (Huang et al., 2021), WO_3_ (Gondal et al., 2017), as well as carbon-based nanomaterials such as GO (Kusworo et al., 2021), g-C_3_N_4_ (Li et al., 2019) or SWCNTs (Jue et al., 2020).

**FIGURE 6:**
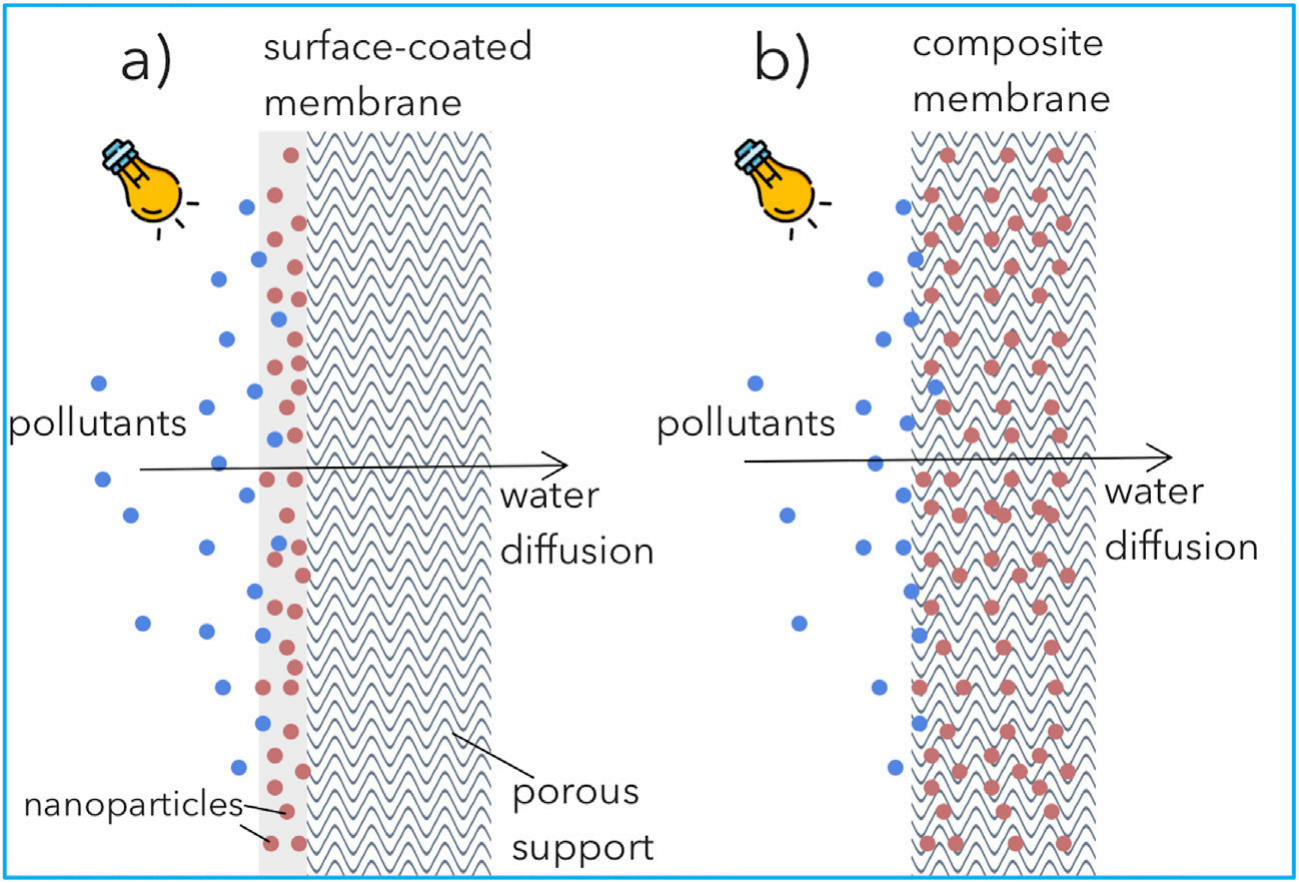
Schematic representation of nanoparticles immobilized in/on composite membranes: **(A)** surface-coated membrane; **(B)** nanoparticles matrix-blended membrane.

Due to high thermal, chemical and mechanical stability, the most used membrane matrix materials are ceramic (Zhang et al., 2020) and polymeric, such as polyvinyl fluoride (PVDF) (Cui et al., 2019), polytetrafluoroethylene (PTFE) (Feng et al., 2018) and polyaniline (PANI) (Vijayakumar and Khastgir, 2018). Nanocomposite membranes can be prepared by nanoparticles surface coating (Figure 6A) or entrapping nanoparticles thru blending with the membrane substrate during the manufacturing process (Figure 6B), or even as a free-standing catalyst (Homocianu and Pascariu, 2022).

Surface-coated membranes can be obtained by dip-coating, electrospraying, sputter deposition or gas-phase deposition which generates a nanoparticles layer on its surface (Lakhotia et al., 2018; Alipour Atmianlu et al., 2021). Horovitz et al. (2016) reported the dip-coating of a commercial alumina membrane with N-doped TiO_2_ by the sol-gel method. Substantial gain in the carbamazepine removal was observed, however, with a loss in the permeability. Recently (Barati et al., 2021), observed that the *in-situ* growth of hydrophilic iron oxides nanoparticles (<4 nm) did not modify the morphology, porosity or the intrinsic hydraulic resistance of ceramic membranes, however, it significantly increased the rejection of humic acid. Higher loading of iron oxides promoted a porous obstruction, thus a drop in permeability.

Matrix-blended membranes obtained by blending nanoparticles in the membrane matrix allow to minimize the possible catalyst leaching. For fabrication, nanoparticles are dispersed by mixing in the polymer-solvent solution before phase inversion. Martins et al. (2016) immobilized 3-8 wt% TiO_2_ nanoparticles in porous PVDF/NaY-based membrane via solvent casting method. Interestingly, the authors reported a minor loss (∼3%) in pollutant removal efficiency compared to the nanoparticle suspension assays. Following a similar strategy, Luo et al. (2021) immobilized NCQDs/BiOBr/TiO_2_ in PVDF membranes with strong antifouling properties under visible light irradiation. The use of blended nanoparticles promoted the degradation of the pollutant while enhancing the filtration performance due to lower membrane fouling. Yet, the membrane permeability can be compromised due to porous hindrance when high nanoparticle loading is used.

In summary, nanoparticles immobilized in membranes is an effective strategy to overcome the disadvantages associated with conventional slurry-type systems. Surface-coated membranes can improve hydrophilicity and provide antifouling properties; however, the strategy can be ineffective in preventing catalyst leaching. Nanoparticles blended in the membrane matrix are more successful in retaining the catalyst, but the antifouling capacity can be inferior. Deeper optimization to assure porosity, surface dispersion, high photocatalytic activity and improve mass transfer is required to enlarge the broad market commercialization of self-cleaning nanocomposite membranes for environmental application.

### 2.4 Nanostructured platforms for SERS detection

Fleischmann and co-workers noticed the SERS effect for the first time in 1974 (Fleischmann et al., 1974), with its interpretation and deep understanding of underlying mechanisms developed in the subsequent years (Albrecht and Creighton, 1977; Jeanmaire and Van Duyne, 1977; Jensen et al., 2008; Stiles et al., 2008; Valley et al., 2013). However, it was mainly during the last decades that SERS has been regarded as an essential method for water quality monitoring, offering new possibilities for practical implementation and, in certain cases, lowering analytical detection limits in established analytical protocols (Pinheiro et al., 2018; Tang et al., 2018; Bodelón and Pastoriza-Santos, 2020; Ong et al., 2020). The increasing interest in the application of SERS to water quality monitoring is inseparable from last developments in terms of instrumentation (e.g., detection sensitivity and portability) and also on the nanofabrication methods of analytical substrates, in which surface chemistry plays a determinant role (Li et al., 2014; Yılmaz et al., 2022). In fact, our interest in this research topic has been mainly driven by exploring chemical routes to produce a variety of nanostructured materials with specific features for SERS analysis (Fateixa et al., 2011; Fateixa et al., 2013; Fateixa et al., 2015; Fateixa et al., 2016; Pinheiro et al., 2019b).

#### 2.4.1 Nanometal assemblies in polymeric substrates

The fabrication of flexible, low-cost and sensitive SERS substrates has gained great attention in environmental monitoring (Yu and White, 2012; Gao et al., 2016; Fateixa et al., 2018b; Guo et al., 2019; Sun et al., 2021). Polymer-based filter membranes show important features for these purposes, namely robust mechanical resistance and tuneable pore size. These membranes are available in various materials that allow sample concentration by large-volume filtration, promoting the target analyte uptake and, consequently, easier detection by SERS (Xu et al., 2021b). The lightweight and flexibility of membrane SERS substrates can also be combined with portable Raman spectrometers and smartphones, providing on-site detection (Gao et al., 2016; Guo et al., 2019; Sun et al., 2021). Several filtering membranes have been used as SERS substrates, such as cellulose (paper) (Lee et al., 2010; Moram et al., 2018; Wu et al., 2018; Jiao et al., 2022), polyamide (PA) (Yu and White, 2012; Shi et al., 2014; Fateixa et al., 2018a; Yu et al., 2021), PVDF (Yu and White, 2012; Gao et al., 2016; Guo et al., 2019; Sun et al., 2021), and derived polymer-based composites (Fateixa et al., 2018b; Ankudze et al., 2019; Ye et al., 2020). Polymer-based membranes offer flexibility, porosity and extensive fibrillar networks for surface chemical functionalization, which are important features for substrates fabrication, but they are not SERS active themselves. In this regard, plasmonic nanoparticles such as Ag or Au NPs are often used as the SERS active phases present in the filter membranes. The attachment of metal NPs onto the filter membranes can be performed by different approaches, such as 1) filtering colloidal NPs through the membrane (Yu and White, 2012; Shi et al., 2014; Fateixa et al., 2018a); 2) blending or *in situ* growth of NPs within the filter membrane (Lee et al., 2010; Moram et al., 2018; Wu et al., 2018; Yu et al., 2021); 3) coating the filter membrane with Au/Ag assembled structures (Guo et al., 2016); 4) modification of the fibres with NPs prior the membrane fabrication (Guo et al., 2016; Fateixa et al., 2018b; Ankudze et al., 2019).

A straightforward method for SERS substrates based on filter membranes involves syringe filtration of metal sols, as demonstrated by the early work of Yu and White. (2012) on Ag colloidal NPs supported on PA and PVDF membranes. They have demonstrated that this simple, economic and fast method provides efficient SERS substrates, which not only pre-concentrate the target analyte but also decrease the analysis detection time, with improvements in the Raman signal reproducibility of the molecular probe used. This topic was further explored in other laboratories using distinct filter membranes and plasmonic NPs with diverse morphologies, such as spherical Ag and Au NPs (Guo et al., 2016; Fateixa et al., 2018b; Sun et al., 2021; Yu et al., 2021), nanorods and nanowires (Lee et al., 2010; Shi et al., 2014; Wu et al., 2018; Ankudze et al., 2019), nanostars and nanourchins (Mehn et al., 2013; Xu et al., 2021a), and Au/Ag alloys or core-shell (Moram et al., 2018; Sha et al., 2020; Khan et al., 2022). Yu et al. (2021) have investigated Au/PA filter membranes for pesticide detection by comparing distinct fabrication methods, including *in situ* reduction, immersion-adsorption, and filtration. The latter method provided the SERS substrates with the best stability, reproducibility, and the lowest detection limit for the selected pesticides, compared with those prepared using other methods. Gao and co-workers have developed a rapid and sensitive methodology to detect the fungicide ferbam in aqueous samples using a portable Raman spectrometer (Gao et al., 2016). In this way, Ag colloids were previously aggregated by electrolyte addition (NaCl) and then filtered through a PVDF filter membrane. The Ag-loaded PVDF membranes allowed the SERS detection of ferbam dissolved in distilled, tap, and pond water samples at a low concentration of 2.5 μg/L.

**FIGURE 7:**
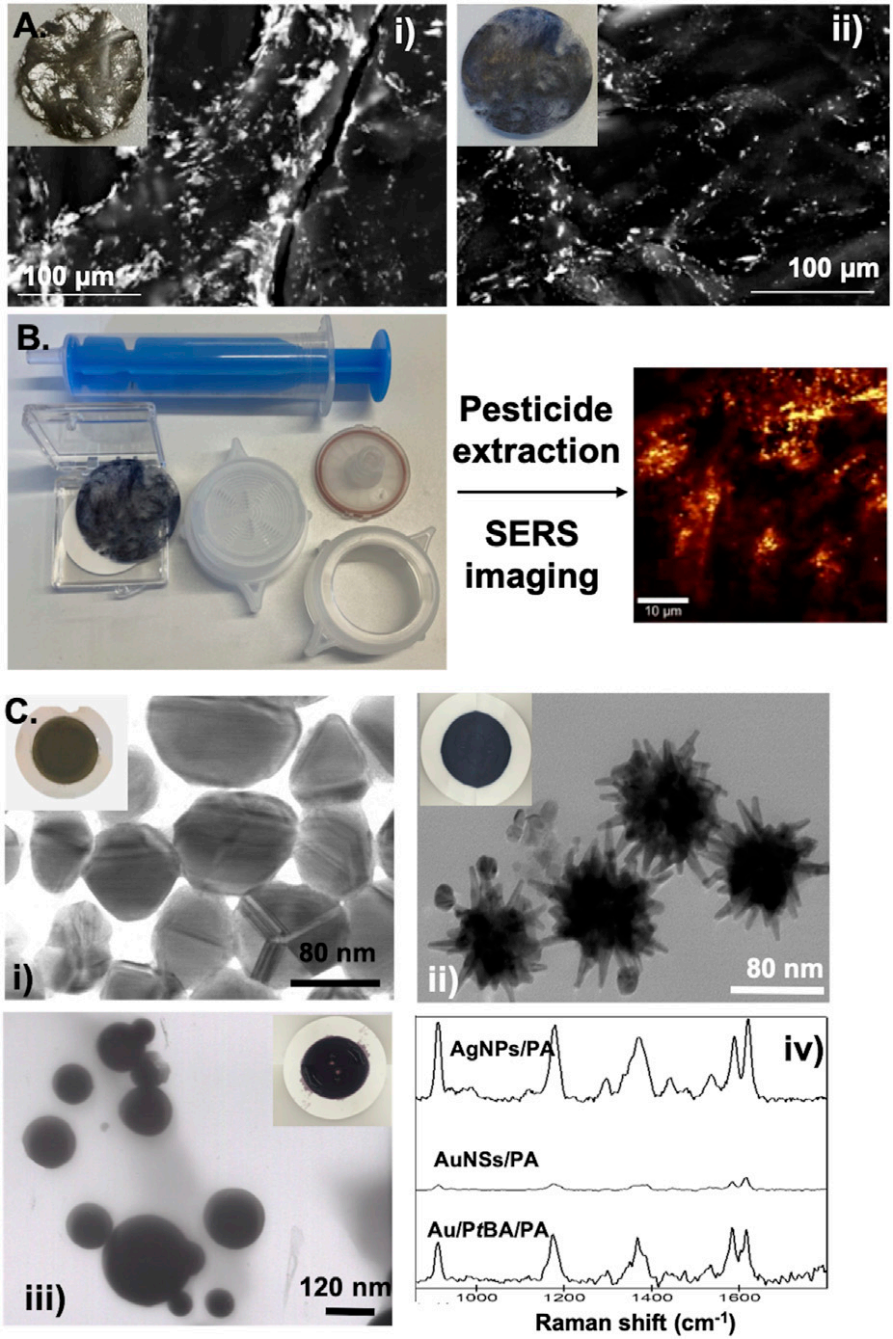
**(A)** SEM images of Ag/LCP (i) and Au/LCP (ii) composites prepared by layer-by-layer approach. Inset: digital photography of the MNPs/LCP fibres after pressed by hydraulic pressing **(B)** Schematic illustration of MNPs/LCP filter membrane preparation for SERS detection of pesticides dissolved in water. **(C)** TEM images of Ag NPs (i), Au nanostars (ii), and Au NPs coated with P*t*BA (iii) deposited on commercial PA filter membranes and used as SERS substrates for the detection of crystal violet (iv). Insets: digital photographs of PA filter membranes loaded with Ag NPs (i), AuNSs (ii) and Au/P*t*BA beads (iii).

Our interest in this topic led us to explore the fabrication of filter membranes using PA and liquid crystal polymer (LCP) for water CEC monitoring (Fateixa et al., 2018a; Fateixa et al., 2018b; Fateixa et al., 2019). A simple strategy was reported to fabricate filter membranes based on composites containing plasmonic NPs and Vectran™, a manufactured aromatic poly (esther) that shows properties useful for filtering membranes (Fateixa et al., 2018b; Fateixa et al., 2019). In fact, Vectran™ is a thermotropic LCP fiber with high Young modulus and high strength, superior heat resistance and excellent chemical stability in comparison to other conventional polymers. Hence, Au and Ag NPs with different morphology (spheres and rods) have been prepared by wet chemical procedures and successfully attached to the LCP fibres previously modified with polyelectrolytes. In this strategy, the optical properties are fine-tuned through judicious selection of the metallic NPs which have been prepared previous the composite fabrication. The metal-loaded nanocomposites were then submitted to hydraulic pressing to fabricate the filter membranes. These SERS substrates were investigated to uptake and detect thiram and paraquat dissolved in water samples using SERS coupled with Raman imaging (Figure 7A).

Alternatively, the *in situ* citrate reduction method applied to Ag(I) dissolved in LCP aqueous suspensions resulted in Ag/LCP fibres, which were then pressed into filter membranes. As a proof of concept, the Ag/LCP filter membranes were supported on polyamide filters, which combine the SERS technique and share similarities with solid phase extraction due to the rapid collection of the molecular analyte for *in situ* detection (Figure 7B). These SERS substrates have shown better SERS response and selectivity for thiram molecules dissolved in water samples than other pesticides (e.g., paraquat). The SERS map shows a strong SERS signal of the thiram molecules, indicating not only the pesticide’s spatial distribution but also the active Raman regions (so-called hotspots) due to the presence of the Ag NPs (Fateixa et al., 2018b). These membranes applied to real aqueous matrices laboratorial spiked with thiram led to SERS signals with enhancement factors of 1.67 × 10^7^ and 3.86 × 10^5^, respectively, for Aveiro estuary water and fruit juices samples. The latter value was lower than the maximal residue limit of 5 ppm in fruit, as prescribed by European regulations (EU) 2016/1.

Other studies involved metal-loaded PA filter membranes fabricated by filtration under reduced pressure to detect crystal violet, a synthetic basic cationic dye used in veterinary medicine to treat fungal infections in aquacultures (Fateixa et al., 2018a; Fateixa et al., 2019). Commercial PA filter membranes with a porous size of 200 nm are suitable platforms to load metallic NPs. We have succeeded in depositing Ag and Au particles with different particle sizes and shapes (spheres and stars), and polymeric coated Au NPs (Au/poly (*tert*-butyl acrylate)) and used them as SERS substrates (Figure 7C). Using these metal-loaded PA filter membranes, we have reported, for the first time, the use of Raman imaging combined with SERS spectroscopy not only to optimize the fabrication process of such SERS substrates but also to achieve low detection limits. In more detail, SERS imaging was used to adjust operational parameters, namely the amount of NPs in the filter membrane surface and sample preparation method, and to monitor the active SERS sites’ formation in distinct areas of the PA membrane. The Ag/PA filter membranes led to the higher SERS response in the CV analysis with detection limits of 10 fM for Aveiro Estuary water (Fateixa et al., 2018a). This fabrication process can be easily scaled up, and large amounts of contaminated water can be used for in field measurements.

#### 2.4.2 Inkjet printing of SERS substrates

Several methods for fabricating SERS substrates rely on the adaptation of available technologies for routine applications. Among such methods, inkjet printing has also been explored to produce substrates for SERS detection of water pollutants (Yu and White, 2010; Dai et al., 2014; Restaino et al., 2017; Godoy et al., 2020; Martins et al., 2021). This technique has several advantages such as simplicity, low-cost and suitability for large-scale production of substrates. Furthermore, it offers the possibility to adjust the SERS sensor to target applications by employing ink formulations of distinct chemical composition (Yorov et al., 2016; Martins et al., 2021; Fernandes et al., 2022). Another important feature of inkjet printing is that allows the fabrication of SERS sensors on diverse materials with different substrate shapes (e.g., cellulose paper, glass surfaces or silicon wafers) (Hoppmann et al., 2013; Yang et al., 2015; Miccichè et al., 2018). In particular, paper is a flexible, biodegradable, lightweight and low-cost material, therefore is highly attractive to develop disposable analytical platforms. Furthermore, paper-based SERS substrates can be employed in point-of-use applications in conjunction with handheld Raman instruments and smartphones (Sun et al., 2019; Zeng et al., 2019). Thus, the inkjet printing of colloidal metal NPs on paper is gaining considerable attention as a widely accessible method for SERS substrates fabrication (Dai et al., 2014; Godoy et al., 2020; Martins et al., 2021; Fernandes et al., 2022).

Earlier reports on the use of cellulose substrates demonstrated their potential for SERS detection using both vegetable cellulose and bacterial cellulose (Marques et al., 2008). The latter has some advantages, because the cellulose can be used either as dry substrates (paper sheets) or aqueous sponges, both with high level of metal loaded cellulosic nanofibrils. However, the demonstration of the inkjet printing method for fabricating SERS paper-based substrates was only reported later by Yu and White. (2010). These authors have used a commercial inkjet printer to pattern Ag NPs with microscale precision on paper to obtain SERS substrates. In this way, they could detect rhodamine 6G (R6G) dissolved in water in a concentration as low as 10 fmol/µL. Later, White et al. have shown that inkjet printed paper substrates are also sensitive for the detection of antibiotics (e.g., sulfapyridine, ciprofloxacin) (Restaino et al., 2017). Many other reports followed that apply the inkjet printing method using chemical and morphological distinct colloids as the inks, such as Au nanospheres (Godoy et al., 2020), Au nanorods (Dai et al., 2014), and Ag anisotropic NPs (Yorov et al., 2016). In fact, it is not an overstatement to consider the chemistry of nanoinks for printing materials a research topic on its own, such as in the fabrication of paper-based SERS substrates, considering the many possibilities to adjust the sensing properties by applying chemical reasoning to the ink composition.

**FIGURE 8:**
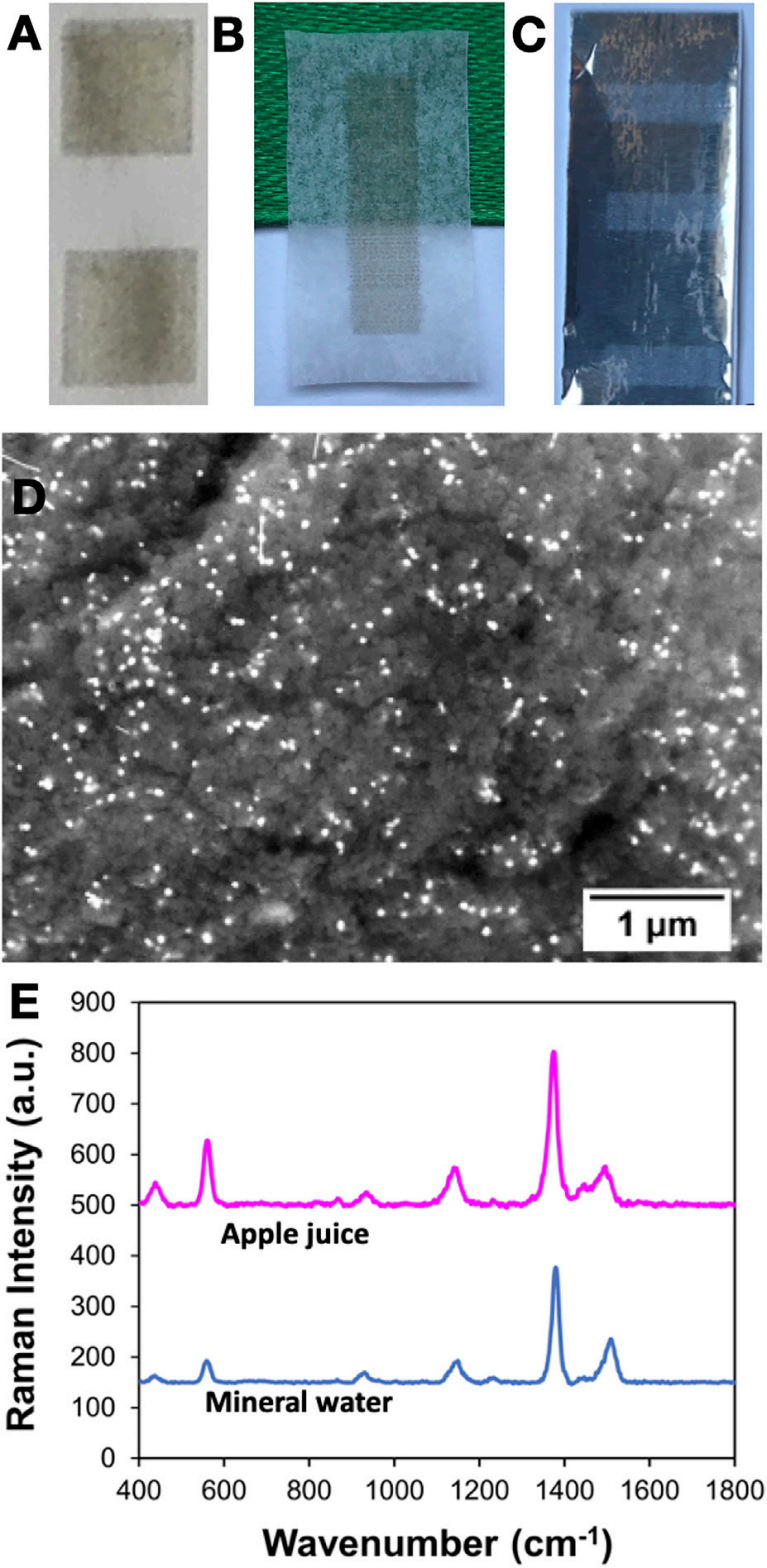
SERS substrates prepared by inkjet printing of metal colloids on different types of paper: **(A)** Ag/PS on office paper; **(B)** Ag on parchment paper **(C)** Au/PS on aluminium foil paper; **(D)** SEM image of office paper printed with Ag/PS ink, showing the metal NPs as white spots on a polymeric background.; **(E)** SERS spectra of thiram (10^−6^ M) spiked in apple juice and mineral water using Ag/PS as the substrate.

We have been particularly interested in ink formulations for the preparation of paper-based SERS substrates that offers control of the surface hydrophobicity (Martins et al., 2021). In fact, it has been demonstrated that hydrophobic paper can improve the sensitivity of SERS sensors by preventing the spreading of aqueous droplets employed in the analysis (Lee et al., 2018; Godoy et al., 2020). As consequence, this strategy can be used for increasing the amount of plasmonic NPs in a small area of the substrate by using the respective hydrosols and also allows the concentration of the aqueous analyte in the sensing region. Figure 8A shows paper-based SERS substrates obtained through inkjet printing of aqueous emulsions containing Ag NPs (Figure 8D), as the SERS active phase, and polystyrene (PS) beads that confers hydrophobic properties to the coated paper. Substrates with different levels of hydrophobicity were obtained by varying the weight percentage of PS in the ink formulations and then evaluated using several aqueous samples. The best SERS performance of the Ag/PS coated papers was observed for substrates showing the highest hydrophobicity. It should be noted that this approach is not limited to common office paper; other substrates can be coated either varying the emulsion composition or the metal colloids used in the inkjet printing process, as illustrated in Figures 8A–C. The applicability of these substrates using different aqueous matrices was evaluated, such as in the SERS detection of thiram (LOD 0.024 pm) in spiked samples of mineral water and apple juice (Figure 8E) (Martins et al., 2021).

More recently, we have reported the use of dendrimer-stabilized Au:Ag nanoalloys of variable molar ratios for the inkjet printing of SERS substrates on office paper (Fernandes et al., 2022). Firstly, Au:Ag alloys were prepared in the presence of poly (amidoamine) dendrimer (PAMAM), resulting in nanoassemblies with plasmonic properties that depend on the chemical composition of the final materials (Fernandes et al., 2021; Fernandes et al., 2022). The dendrimer acts as a “molecular glue” for the clustering of Au and Ag nanocrystals, and also as a reducing and colloidal stabilizer. The dendrimer-stabilized Au:Ag nanoalloys were then used as colloidal inks for the inkjet printing of SERS substrates on paper. To further improve the SERS performance of the substrates, the paper surface was pre-treated with a hydrophobic coating of PS before the deposition of the nanoassemblies. The resulting substrates displayed good SERS sensitivity for the detection of the pesticide thiram in aqueous solutions, namely by using a portable Raman equipment.

#### 2.4.3 Magneto-plasmonic substrates

**FIGURE 9:**
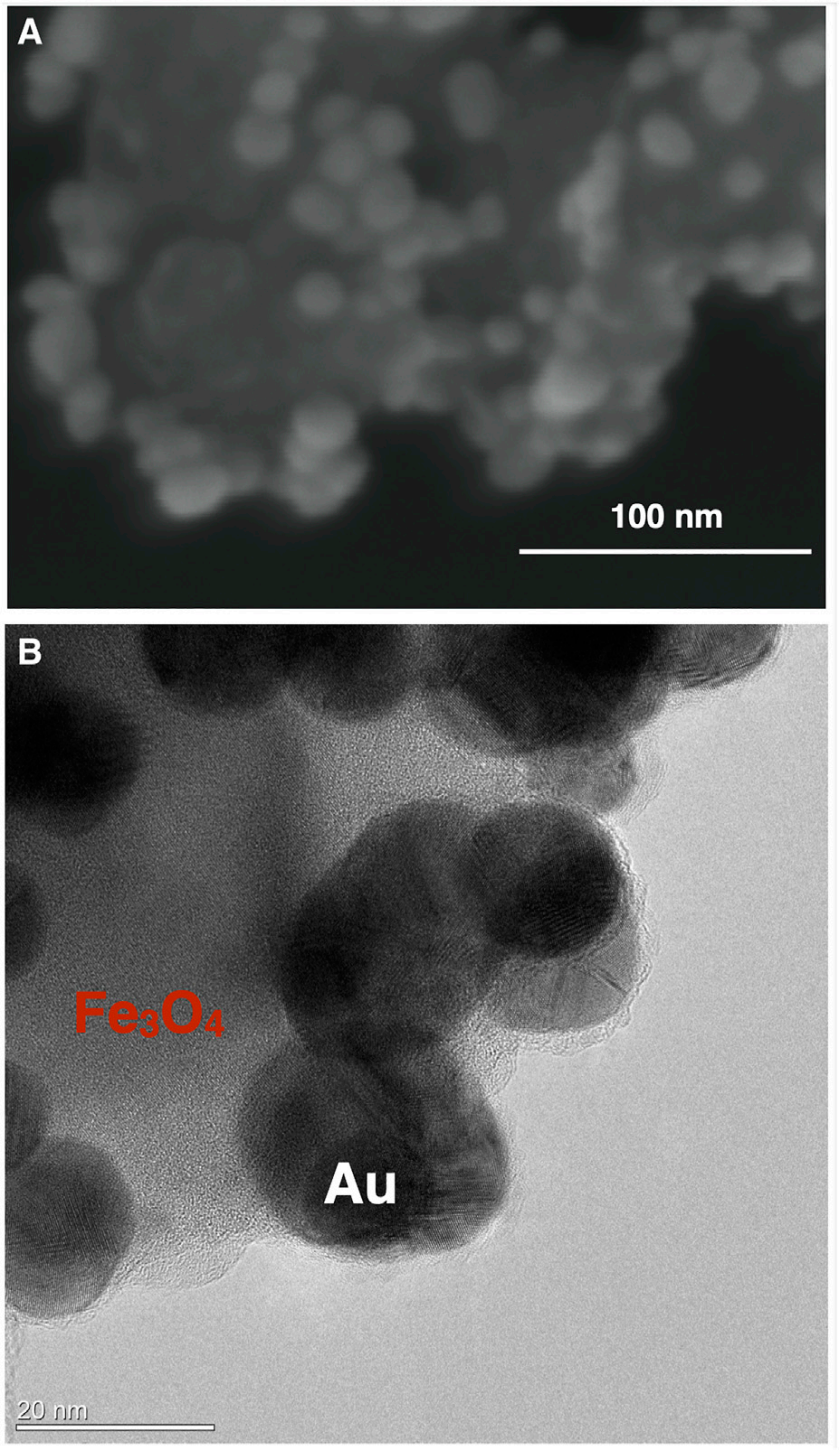
Electron microscopy images of multifunctional magneto-plasmonic nanosorbents: SEM **(A)** and TEM **(B)** images. The magnetic drivers are Fe_3_O_4_ particles decorated with SERS active Au nanoparticles.

Owing to their multifunctionality, there has been great interest in magneto-plasmonic systems where two distinct inorganic phases coexist in the same nanostructure, such as in a composite particle of a plasmonic metal and a magnetic iron oxide. These nanostructures can be produced by a variety of methods, leading to a wide assortment of magneto-plasmonic systems. In this regard, we have been interested in colloidal procedures because they can be easily implemented by adaptation of synthesis protocols developed for the single-component counterparts. Hence, two main synthetic approaches have been investigated: 1) the assembly of colloids of both phases and 2) the *in situ* chemical reduction of metal salts in the presence of the colloidal magnetic phase; the latter are typically ferrimagnetic Fe_3_O_4_ NPs. Although featuring distinct characteristics, both methods result in magneto-plasmonic nanosorbents whose basic configuration is observed in the electron microscopy images shown in Figure 9 (Lopes et al., 2016; Pinheiro et al., 2018). In Figures 9B, a detailed TEM image of Fe_3_O_4_-Au nanosorbents show Au spheroidal NPs (ca. 15 nm) assembled onto surface modified Fe_3_O_4_ NPs (ca. 80 nm); the Au NPs are responsible for SERS activity while Fe_3_O_4_ allows the separation of the multifunctional particles by applying an external magnetic gradient. The attachment of the Au NPs followed previous surface modification of the Fe_3_O_4_ particles with silica shells enriched in dithiocarbamate moieties by using a single-step route (Tavares et al., 2013). Hence, the modification of the nanosorbents’ surfaces is a practical consequence of the concept of soft acid (Au)-soft base (S ligand) affinity, which can be extended to the attachment of other noble metal NPs onto dithiocarbamate functionalized magnetites (Lopes et al., 2016).

There are few reports in the literature on the development of colloidal magneto-plasmonic nanomaterials applied to environmental applications, though their multifunctionality have been explored in other areas such as in bioapplications (Hao et al., 2010; Hou et al., 2017; Nguyen et al., 2018; Ovejero et al., 2018; Scaramuzza et al., 2019; Archana et al., 2021; Mukha et al., 2021). However, nanostructures like those shown in Figure 9 can be also explored as a new class of multifunctional nanosorbents for analytical purposes. While the magnetic iron oxide NPs confer the ability for magnetic separation from the aqueous medium, the plasmonic metal NPs allow the SERS monitoring of the target analyte. In this context, we have reported the application of Fe_3_O_4_-Au nanosorbents in the uptake and SERS detection of water contaminants of emergent concern, such as vestigial pharmaceutics (Pinheiro et al., 2019a; Pinheiro et al., 2019b). These multifunctional nanosorbents have great potential in analytical kits envisaging water quality monitoring protocols to be applied both in laboratory and on-site analysis. A great advantage in using magneto-plasmonic nanosorbents for trace analysis is the possibility of successive pre-concentration steps to facilitate the detection of the analyte. Nevertheless, research on this topic has been scarce and the potential of magneto-plasmonic materials is far from being well explored.

### 2.5 Environmental impact of nanomaterials

To overcome the global problem of water pollution, nanomaterials emerged as promising tools for monitoring water quality and for the remediation of wastewaters (Vikesland, 2018; Yaqoob et al., 2020; Davarazar et al., 2021). This is because some of their properties like nano size, large specific surface area, high reactivity, porosity, among others, can be explored for more effective processes. But, despite the beneficial potentials conferred by these properties for water and wastewater monitoring and remediation, these same properties may render them toxic to the biota (Nogueira et al., 2016; Monteiro et al., 2019; Costa et al., 2020; Ghadimi et al., 2020). Considering that it is inevitable that some of the nanoparticles used in water and wastewater treatments will be released into the aquatic ecosystem, concerns arise on their ecotoxicity to the environment. Therefore, targeting the safe use of nanomaterials in the remediation of water and wastewaters and promote the development of sustainable remediation nanotechnologies, in parallel to the study of its chemical efficiency (i.e., removal and/or monitoring of contaminants in water), it is important to address if the nanoparticles per se may constitute a risk to the environment. We have carried out this type of studies as exemplified by the case of study presented below.

**FIGURE 10:**
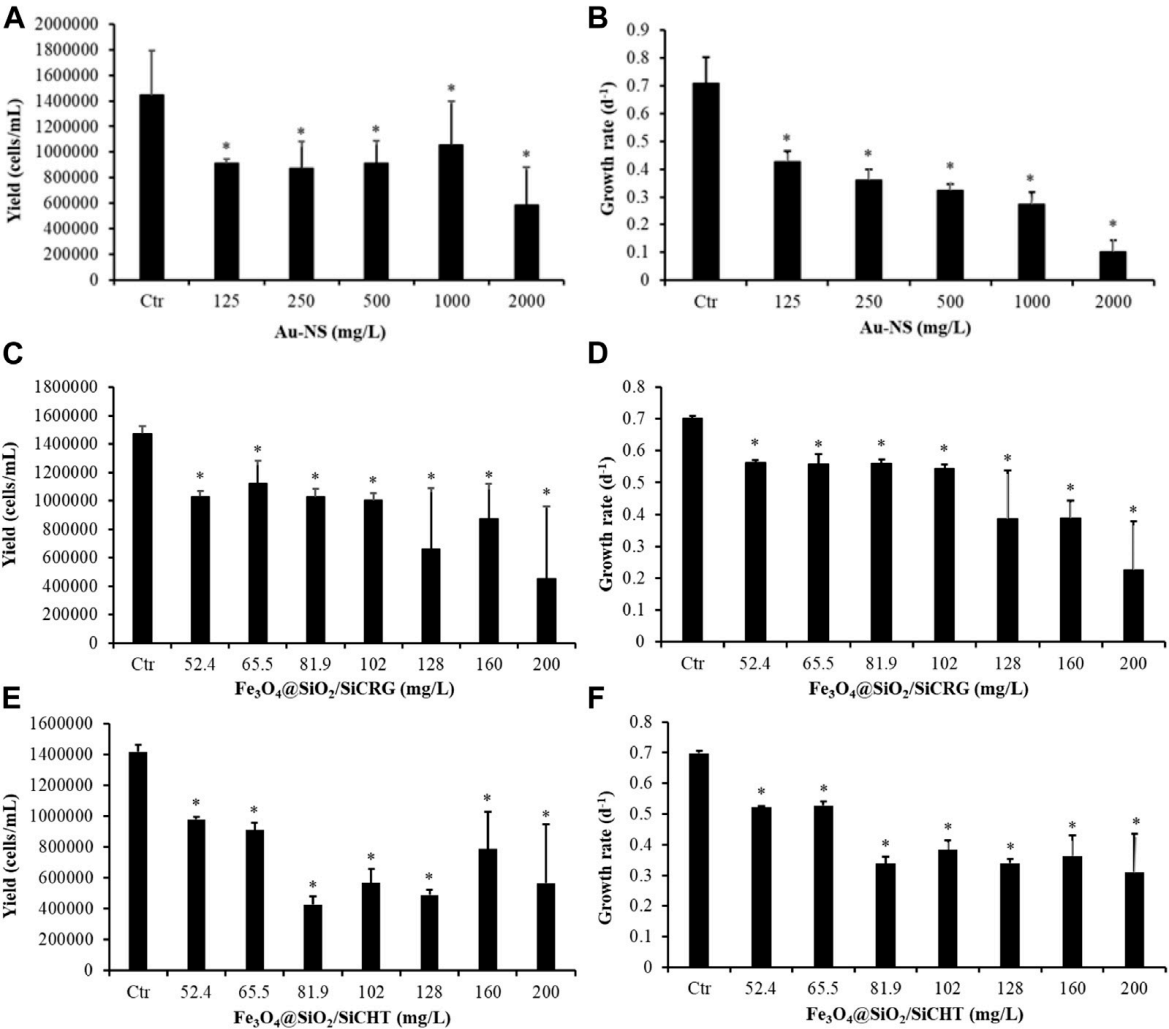
Average yield and growth rate of *Raphidocelis subcapitata* after being exposed, for 72 h, to several concentrations of gold nano-speres (**(A)** and **(B)** Au-NS) and of Fe_3_O_4_ nanoparticles coated with κ-carrageenan hybrid siliceous shells (**(C)** and **(D)** Fe_3_O_4_@SiO_2_/SiCRG) and Fe_3_O_4_ nanoparticles coated with chitosan hybrid siliceous thin shells (**(E)** and **(F)** Fe_3_O_4_@SiO_2_/SiCHT). * Indicates significant differences from the respective control (*p* < 0.05).

We have characterized the ecotoxicity of three systems, mentioned previously in this manuscript, to the freshwater microalgae *Raphidocelis subcapitata*. The selected systems comprised colloidal gold nanostars (Au-NS) and biocomposite magnetic nanosorbents. The latter samples have distinct surface chemistry, one sample comprise coatings of k-carrageenan hybrid siliceous shells (Fe_3_O_4_@SiO_2_/SiCRG) and the other contains chitosan hybrid siliceous thin shells (Fe_3_O_4_@SiO_2_/SiCHT). In particular, the selected biocomposite sorbents are of particular interest because they have shown capacity to remove CEC from aqueous media as discussed above (Soares et al., 2016; Soares et al., 2017a). The *R. subcapitata* was used here as the model species because it is one of the recommended by REACH legislation to be tested when characterizing the ecotoxicity of new chemicals during the process for authorization to be placed in the market. Therefore, 72-h growth inhibition assays were performed by exposing *R. subcapitata* to several concentrations of Au-NS (125–200 mg/L), Fe_3_O_4_@SiO_2_/SiCRG (52.4–2000 mg/L) and Fe_3_O_4_@SiO_2_/SiCHT (52.4–2000 mg/L). Figure 10 apresents the results obtained in these assays. All the tested concentrations of the three selected nanomaterials induced a significant decrease in the yield and growth rate of *R. subcapitata* (*p* < 0.05). Regarding Au-NS, the concentration causing 20% (EC_20_, considered as the threshold for significant effects) and 50% (EC_50_) of reduction in the growth rate were 32.3 (95% confidence limits-95%CL: 8.84–7.4) and 294.8 (95%CL: 134.4–455.1 mg/L), respectively. Considering these ecotoxicity results, the safe use of Au NS depends on the amount employed for water quality monitoring applications. For example, in a study on magnetite decorated with Au-NS for SERS detection of tetracycline, only 0.250 mg/L of Au-NS were necessary (Pinheiro et al., 2019b), thus it is foreseen a safe use of these Au-NS in this context.

The two samples of magnetic biosorbents exhibited similar toxicity to the microalgae (Figure 10), with EC_20_ and EC_50_ of 84.8 (95% CL: 50.4–1119) mg/L and 164 (95% CL: 131-198) mg/L for Fe_3_O_4_@SiO_2_/SiCRG and of 29.7 (95% CL: 7.55–51.9) and 139 (93.8–183) mg/L for Fe_3_O_4_@SiO_2_/SiCHT, respectively. The EC_x_ values computed for Fe_3_O_4_@SiO_2_/SiCRG were lower than the concentrations used by (Soares et al., 2016) to remove metoprolol from water. These authors used a concentration of 500 mg/L of Fe_3_O_4_@SiO_2_/SiCRG (by adding 10 mg of the sorbent to 2 ml of a metoprolol solution) to remove metoprolol from water and reported a removal higher than 30% after 2 h contact time. This removal efficiency occurred at concentrations of sorbent that may induce toxicity to freshwater biota, since it is higher than the concentration that impairs in 20 and 50% the growth rate of *R. subcapitata*. Given that microalgae are at the base of the trophic level, cascade effects at upper trophic levels may be expected to occur. A direct comparison of the EC_x_ here computed for Fe_3_O_4_@SiO_2_/SiCHT could not be done with the amount needed to remove the non-polar compounds (Soares et al., 2017a) because different units were used in the two studies.

The preliminary results on the ecotoxicity of these three systems, suggest the potential for the safe use of Au-NS in the monitoring of water quality. For Fe_3_O_4_@SiO_2_/SiCRG it is expected that the amounts needed for an efficient remediation of contaminated waters will cause an ecological risk to microalgae, suggesting the need to remove these nanosorbents from the treated water before being released into the natural aquatic systems. In this regard, the ability for magnetic separation offered by these nanosorbents constitutes a good asset but that still require further research. In fact, it must be emphasized that these experiments were performed under controlled laboratory conditions and natural waters are much more complex systems than the aqueous media used in laboratory, which may alter the toxicity observed under laboratory conditions. Therefore, these types of studies should also be carried out under more realistic scenarios (e.g., test in media that are relevant/similar to the ones aimed to be remediated) to better understand how the nanosorbents will behave in natural freshwaters. Moreover, scientific evidence exists reporting how environmental characteristics may affect the fate and behaviour of nanoparticles, which, in turn, influences their toxicity to biota [e.g., (Ren et al., 2016; Arvidsson et al., 2020) and references therein]. Furthermore, the toxicity of these nanoparticles after being aged in the environmental matrices must be also explored since published works have reported a reduction in their ecotoxicity after ageing in water and soil matrices (Lehutso et al., 2020).

## 3 Conclusions and outlook

This review focused on recent research on nanomaterials of colloidal nature, to be used in this form or as starting components for other types of materials, envisaging applications that aim to prevent contamination of water sources and/or their treatment, mainly when CEC are involved. The revision of scientific work presented here was also framed by the research topics that our own group has developed on this subject. The several examples presented demonstrate the enormous potential of colloidal nanomaterials in applications aimed at water quality improvement. However, they are in distinct stages of potential application and, in certain cases, mainly regarded as interesting systems for acquiring new scientific knowledge. This is due not only to the nature of nanomaterials and their differentiated complexity, but also to the requirements that apply to each of the technologies in which nanomaterials are used. As an example, it should be noted that the scale at which nanomaterials are used differs between an application as nanosorbent in a water treatment plant and an analytical kit for laboratorial monitoring a target contaminant.

Although research on nanomaterials as addressed in this work is in different stages of maturation, it is possible to highlight some challenges and possible lines of investigation aimed at their solution. Nanosorbents have clear advantages over conventional materials, however aspects related to selectivity for CEC molecules and subsequent regeneration, if necessary, remain important challenges. The application of colloidal materials in photocatalysis has focused mainly on the use of the UV region of the electromagnetic spectrum, thus complementary and innovative strategies are needed aimed at extending the photocatalytic activity to the visible region. The study of chemical speciation of solutions submitted to photocatalytic treatment is of increasing importance, because total mineralization of pollutant molecules has not been always demonstrated. The use of nanomaterials as adsorbents and in photocatalysis, in a real context of water cleaning technology, must be preceded by environmental impact studies. The strategy of associating magnetic phases either to adsorbents or photocatalysts, is a possible solution to ensure the development of safe and more sustainable processes based on magnetic separation technologies. Another alternative strategy could be the immobilisation of nanomaterials in membranes using new processing technologies that take advantage of the properties of colloidal nanoparticles. With regard to the development of SERS sensors for CEC detection, in addition to the detection limit and selectivity for target analytes, there is a need for quantitative analytical data for specific scenarios. This aspect requires even more fundamental research that integrates, in addition to the aspects of synthesis of plasmonic nanomaterials, the mechanisms associated to their interaction with the analyte molecules, namely by taking into account the nanoscale characterization of the substrate, which impacts on the uniformity and reproducibility of the SERS signal.

In addition, there is a need for pilot projects aimed at water improvement applications for the most promising colloidal nanomaterials, in a realistic context, by selecting specific situations in which these materials can be part of the solution, in whole or in part. This type of approach also requires further ecotoxicity studies, also under realistic conditions, that allow the safe use of nanomaterials for the specific application for which they are intended and, in the required quantities. Finally, it should be noted that the unique properties observed in some of the nanomaterials reviewed here, can also be explored in other research contexts and innovative applications.

## Abbreviations

AOX, adsorbable organic halides; CEC, contaminant of emerging concern; GNP, graphite-like nanoplatelets; GO, graphene oxide; ICPTES, 3-isocyanatopropyl triethoxysilane; LCP, liquid crystal polymer; MF, microfiltration; NF, nanofiltration; NP, nanoparticle; PA, polyamide; PANI, polyaniline; POM, polyoxometalate; PS, poly(styrene); PTFE, poly(tetrafluoroethylene); PVDF, poly(vinyl fluoride); rGO, reduced graphene oxide; RhB, Rhodamine B; SDG, Sustainable Development Goal; SEM, Scanning electron microscopy; SERS, surface enhanced Raman scattering; UN, United Nations; SWCNT, single-walled carbon nanotube; TCE, technology-critical element; TEM, Transmission electron microscopy; TEOS, tetraethylorthosilicate; UF, ultrafiltration.
