# Corrigendum: Colloidal nanomaterials for water quality improvement and monitoring

**DOI:** 10.3389/fchem.2023.1209263

**Published:** 2023-04-28

**Authors:** Ana C. Estrada, Ana L. Daniel-da-Silva, Cátia Leal, Cátia Monteiro, Cláudia B. Lopes, Helena I. S. Nogueira, Isabel Lopes, Maria J. Martins, Natércia C. Martins, Nuno P. F. Gonçalves, Sara Fateixa, Tito Trindade

**Affiliations:** ^1^ Department of Chemistry, CICECO-Aveiro Institute of Materials, University of Aveiro, Aveiro, Portugal; ^2^ Department of Biology, CESAM-Centre of Environmental and Marine Studies, University of Aveiro, Aveiro, Portugal

**Keywords:** colloids, nanomaterials, surface chemistry, inorganic nanoparticles, water quality

In the original article, there was an error in the **Funding Statement** on page 17. The reference of the project BIOMAG was incorrectly displayed as “CENTRO-01-0247-FEDER-181268". The correct **Funding Statement** appears below.

“Funding

This work was financed by Portugal 2020 through European Regional Development Fund (ERDF) in the frame of CENTRO2020 in the scope of the project BIOMAG, CENTRO-01-0145-FEDER-181268 and in the scope of the project CICECO–Aveiro Institute of Materials, UIDB/50011/2020 & UIDP/50011/2020 & LA/P/0006/2020, and CESAM-UIDB/50017/2020+ UIDP/50017/2020 + LA/P/0094/2020, financed by national funds through the FCT/MEC (PIDDAC). The work was also partially funded by Portugal 2020 through the European Regional Development Fund (ERDF) in the frame of Operational Competitiveness and Internationalization Program (POCI) in the scope of the project PROTEUS-POCI-01-0247-FEDER-017729. CM and MM thank the Fundação para a Ciência e Tecnologia (FCT) for the PhD grants PD/BD/150568/2020 and SFRH/BD/131433/2017, respectively. NG acknowledge the funding from the European Union’s Horizon 2020 research and innovation programme under the Marie Skłodowska-Curie grant agreement No 101065059. AE, NM, and SF acknowledge the costs of their research contracts (REF-078-88-ARH/2018; REF-069-88-ARH/2018) resulting from the FCT hiring funded by national funds (OE), through FCT, I. P, in the scope of the framework contract foreseen in 4, 5, and 6 of article 23 of the Decree-Law 57/2016, of 29 August, changed by the law 57/2017, of 19 July. CL acknowledges the funding by FCT through CEECIND/03739/2021.”

The authors apologize for this error and state that this does not change the scientific conclusions of the article in any way. The original article has been updated.

